# Annular epidermolytic ichthyosis: a case report and literature review^☆^^☆☆^

**DOI:** 10.1016/j.abd.2019.09.030

**Published:** 2020-05-05

**Authors:** Emanuella Stella Mikilita, Irina Paipilla Hernandez, Ana Letícia Boff, Ana Elisa Kiszewski

**Affiliations:** Dermatology Service, Santa Casa de Misericórdia de Porto Alegre, Universidade Federal de Ciências da Saúde de Porto Alegre, Porto Alegre, RS, Brazil

**Keywords:** Hyperkeratosis, epidermolyitic, Ichthyosis, Ichthyosiform erythroderma

## Abstract

Annular epidermolytic ichthyosis is a rare subtype of epidermolytic ichthyosis that is characterized by erythematous, polycyclic, and migratory scaly plaques accompanied by palmoplantar keratoderma. This report presents the case of an 8-year-old girl who developed migratory, erythematous, scaly plaques associated with palmoplantar keratoderma. The initial hypothesis was erythrokeratodermia variabilis et progressiva; however, the finding of epidermolytic hyperkeratosis in histopathological examination led to the diagnosis of annular epidermolytic ichthyosis.

## Introduction

Annular epidermolytic ichthyosis (AEI) is a rare phenotypic variant of epidermolytic ichthyosis (EI), also known as congenital bullous ichthyosiform erythroderma, an autosomal dominant disorder characterized by extensive erythroderma and formation of blisters in early life.^1^, ^2^, ^3^ Unlike EI, in AEI the clinical symptoms improve in the first years of life, and patients develop annular polycyclic, hyperkeratotic, and erythematous plaques with migratory features, in the trunk and extremities along with palmoplantar keratoderma.^1^, ^4^

## Case report

**Figure 1:**
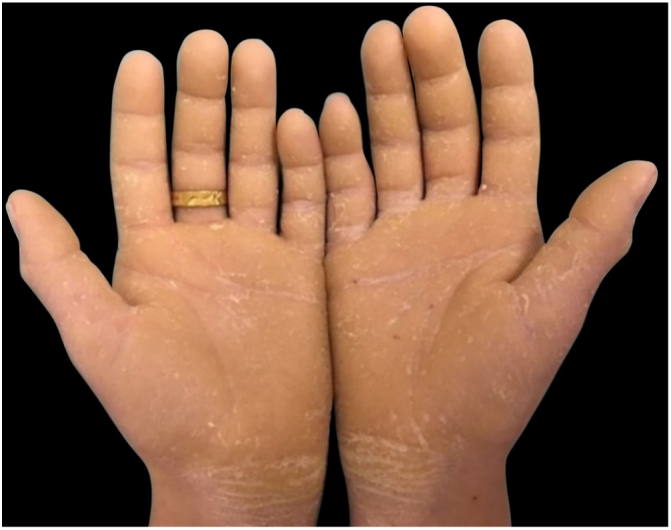
Hyperkeratosis in palms.

**Figure 2:**
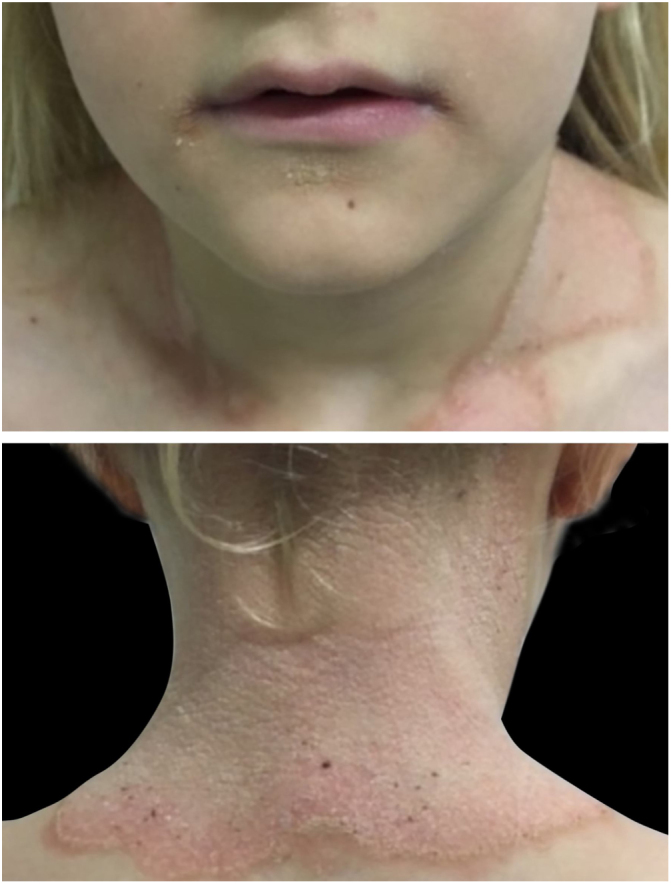
Erythematous, hyperkeratotic plaque, with prominent and geographical border in the cervical region. Yellowish keratotic plaques at the angle of the mouth and chin.

**Figure 3:**
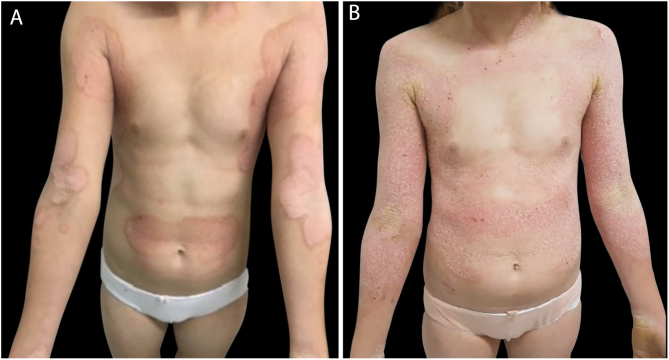
(A) Erythematous, keratotic plaques with prominent and geographical borders on the arms, forearms, cervical, armpits, lateral region of the trunk, and umbilical and supra-umbilical regions. (B) The same patient a month later, presenting erythematous and hyperkeratotic plaques along the entire arm, forearm, and anterior chest, sparing the umbilical and supra-umbilical regions.

An 8-year-old female patient was attended at the pediatric dermatology outpatient clinic of the Hospital Santa Casa of Porto Alegre with a report of diffuse dermatosis that began in the first months of life. Upon examination, the patient presented erythematous, scaly, hyperkeratotic plaques with prominent borders, affecting the mesogastrium, cubital fossae, popliteal fossae, and inguinal and cervical regions, as well as palmoplantar hyperkeratosis and yellowish hyperkeratotic plaques on the scalp and nasal introitus (Figure 1, Figure 2, Figure 3). No associated changes in hair, nails, or mucosa were observed.

The patient denied any symptoms and there was no background of family history of similar cases and consanguinity. After one month, she was reassessed and the appearance of the plaques changed, increasing the extension of the affected areas; however, the polycyclic aspect disappeared (Fig. 3B).

**Figure 4:**
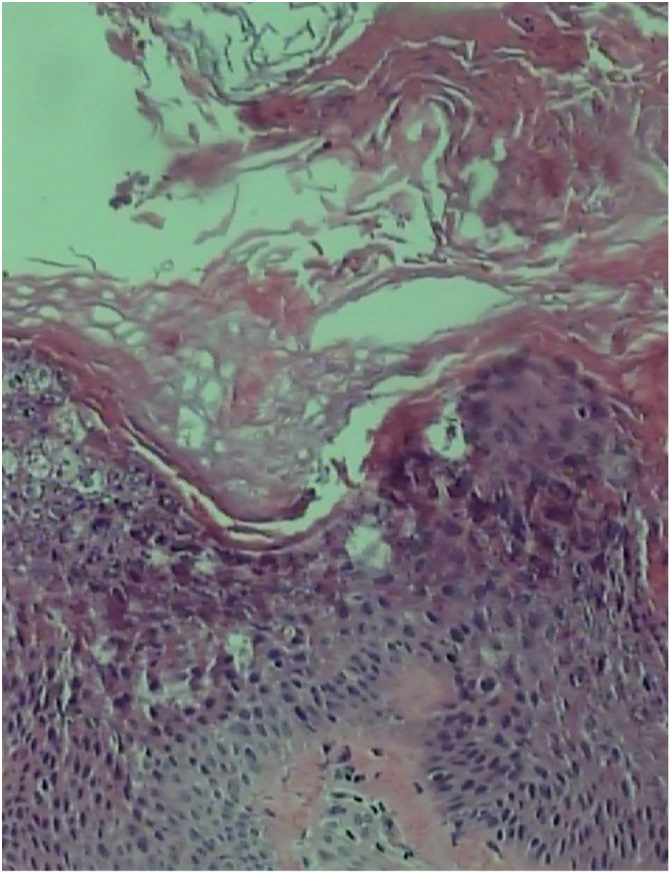
Acanthosis, papillomatosis, and hyperkeratosis with epidermolysis of the granular layer (Hematoxylin & eosin, ×400).

Skin biopsy in the extensor portion of the forearm showed acanthosis, papillomatosis, and hyperkeratosis with marked epidermolysis in the granular layer (Fig. 4).

## Discussion

AEI was first described in 1992 by Sahn et al.^5^ and is the result of dominant mutations in the keratin 1 and keratin 10 genes.^1^, ^2^, ^3^, ^4^, ^5^, ^6^, ^7^ Individuals with this variant may present with bullous ichthyosis at birth and hyperkeratotic lichen plaques on the areas of flexion and extensor surfaces in the first years of life.^1^ Characteristically, they also develop recurrent outbreaks of annular, polycyclic, erythematous, and scaly plaques on the trunk and proximal extremities.^1^, ^4^ The present authors have reviewed the literature published in English, Portuguese, and Spanish since its description and found 19 cases in 10 publications that are summarized in table 1.^1^, ^2^, ^3^, ^4^, ^5^, ^6^, ^7^, ^8^, ^9^, ^10^

**Table 1:** Reported cases of annular epidermolytic ichthyosis.

Authors	Case number	Diagnosis age/sex	Age of onset and clinical presentation	Treatment performed
Sahn et al., 1992	1	30years/F	At 8 months of age, she presented severe and intermittent scaly lesions associated with blisters. In adolescence, she presented hyperkeratotic plaques in the flexures and joints. At the age of 27, she had intermittent annular hyperkeratotic, erythematous, polycyclic plaques on the trunk and proximal region of the limbs, associated with mild palmoplantar keratoderma with fissures.	Topical use of 60% propylene glycol and 6% salicylic acid. Partial response to treatment.
	2	2years/M (son)	At 6 months of age, he developed papules that developed into boli and crusts. At 2 years of age, he had keratotic plaques on his neck and armpits, in addition to multiple erythematous papules on the trunk, extremities, and ear. No report of palmoplantar keratoderma.	

Joh et al., 1997	3 (father)	33years/M	Blisters disseminated from birth. From the 1st year of life, intermittent hyperkeratotic and erythematous plaques associated with blisters and itching up to 16 years of age. At the age of 31, he again presented intermittent blisters and hyperkeratotic plaques in the flexures, ankles, and back of the hands associated with annular migratory, polycyclic, scaly, and erythematous plaques in the trunk and proximal region of the limbs. Palmoplantar keratoderma was not reported.	Oral acitretin 20 mg per day topically associated with ointment containing propylene glycol and 5% urea. Good response to treatment.
	4 (daughter of case 3)	2nd day of life/F	Generalized bullous lesions and moderate erythema since the 2nd day after birth. At 8 weeks of age, she had erosions in the groin and inner thighs associated with mild desquamation on the dorsum of the feet. No report of palmoplantar keratoderma.	Unreported.

Suga et al., 1998	5	11years/M	No history of blisters. Since the age of 7 months, xerotic plates in the flexures and extensor regions. Intermittent episodes of annular, erythematous, scaly, and serpiginous plaques on the trunk and flexures at 11 years of age. No report of palmoplantar keratoderma.	Unreported

Michael et al., 1999	6–9 (members of same family)	NA	Transient blisters from birth. Intermittent erythematous plaques, non-migratory polycyclic hyperkeratotic plaques on the trunk associated with palmoplantar keratoderma.	Topical use of corticosteroids, calcipotriene, tazarotene. and salicylic acid. Minimum response to treatment.

Sybert et al., 1999	10	5years/M	The first hours of life showed bullous lesions and erosions. Since the 1st month, he had intermittent palmoplantar thickening and erythroderma. At 3 years of age, migratory and intermittent, hyperkeratotic, erythematous plaques on the chest, back, and flexures.	Unreported.
	11 (mother of case 10)	18 years/F	Intermittent and migratory erythematous plaques. Erythema and skin erosions since birth. Intermittent palmoplantar hyperkeratosis. Intermittent episodes of erythroderma and palmoplantar keratoderma.	
	12 (aunt of case 10)	NA/F		

Yoneda et al., 1999	13 and 14 (mother and son)	48years/F	Blisters disseminated after birth in both cases with spontaneous improvement in the first months and worsening at around 4 years of age, with annular hyperkeratotic and migratory plaques in the trunk and extremities, maintaining this condition intermittently until adulthood. No report of palmoplantar lesions.	Etretinate without improvement (did not report dose).
		18years/M		

Naik et al., 2003	15	21years/F	No reports of cutaneous lesions at birth. Since the 1st year of life, there were intermittent and migrating polycyclic erythematous plaques on the trunk and hyperkeratotic, verrucous, brownish plaques on the knees, elbows, and ankles. Intermittent palmoplantar keratoderma.	Oral isotretinoin, topical glucocorticoids, and keratolytics. Topical tazarotene with partial response.

Jha et al., 2015	16	26years/F	Spreading blisters, recurrent since birth. Intermittent hyperkeratotic plaques on the trunk from the 1st year of life to 8 years of age. She had hyperkeratotic plaques in the flexures from childhood to adulthood. After the age of 23, she had annular, intermittent, hyperkeratotic, scaly erythematous plaques on the trunk and thighs. No report of palmoplantar keratoderma.	Acitretin 0.5 mg/kg/day. Good response four weeks after starting treatment.
	17	2nd day of life/F	Erythroderma at birth. In the first months of life, hyperkeratotic, polycyclic, scaly plaques on the trunk and limbs. No report of palmoplantar keratoderma.	Use of emollients.

Abdul-Wahab et al., 2016	18	25years/F	No history of lesions at birth. History of ichthyosiform lesions in the knees, elbows, and flexures in childhood. At 25 years of age, erythematous, scaly, and migratory plaques on the trunk and limbs. No report of palmoplantar lesions.	Low dose oral isotretinoin with good response.

Zaki et al., 2018	19	5years/F	Blisters disseminated after birth. Migratory and intermittent erythematous plaques, in addition to hyperkeratotic plaques on the trunk and flexures. Hyperkeratosis and palmoplantar scaling since the first months of life.	Unreported.

M, male; F, female.

In the histopathology, the hyperkeratotic lesions of the AEI revealed hyperkeratosis, acanthosis, and a thickened granular layer. Keratinocytes in the spinous layers and superior granulosa of the epidermis demonstrated cytoplasmic vacuolization and prominent keratohyaline granules.^1^, ^2^, ^4^, ^6^ Basal keratinocytes appeared normal, but there was an increase in the number of mitoses. Regarding findings from electron microscopy, there were abnormal keratin filaments in the suprabasal keratinocytes, increase of kerato-hyaline granules in granule layer cells, and perinuclear accumulations of thickened tonofilament that formed an interrupted perinuclear ring.^4^, ^5^

The main differential diagnosis of annular epidermolytic ichthyosis is with erythrokeratodermia variabilis et progressiva (EKVP),^1^, ^2^ an autosomal dominant cutaneous disorder characterized by erythrokeratodermia and migratory erythematous plaques.^1^, ^2^, ^7^ EKVP is typically associated with mutations in the connexins 30.3, 31, and 43 (GBJ4, GJB3, and GJA1), but recent studies suggest genetic heterogeneity. Distinctive features of EKVP include onset during childhood, absence of epidermal fragility, and histology without evidence of epidermolysis.^1^ The major ultrastructural feature of EKVP is a reduction in the number of keratinosomes in the granular layer.^2^

Treatment options in the small number of patients reported included topical medications such as retinoids, topical corticosteroids, propylene glycol, calcipotriene, and keratolytic agents, and little response was observed.^5^, ^8^, ^9^, ^10^ Three articles report good response with systemic retinoids; two articles cite acitretin treatment with good response^6^, ^8^ and another reports good response with low doses of isotretinoin.^7^

## Financial support

None declared.

## Authors’ contributions

Emanuella Stella Mikilita: Approval of final version of the manuscript; conception and planning of the study; drafting and editing of the manuscript.

Irina Paipilla Hernandez: Approval of final version of the manuscript; conception and planning of the study; drafting and editing of the manuscript; collection, analysis, and interpretation of data; critical review of the literature.

Ana Letícia Boff: Drafting and editing of the manuscript; collection, analysis, and interpretation of data.

Ana Elisa Kiszewski: Approval of final version of the manuscript; conception and planning of the study; drafting and editing of the manuscript; collection, analysis, and interpretation of data; intellectual participation in the propaedeutic and/or therapeutic conduct of the studied cases; critical review of the literature; critical review of the manuscript.

## Conflicts of interest

None declared.
